# Reasons for late presentation to HIV care in Switzerland

**DOI:** 10.7448/IAS.18.1.20317

**Published:** 2015-11-18

**Authors:** Anna Hachfeld, Bruno Ledergerber, Katharine Darling, Rainer Weber, Alexandra Calmy, Manuel Battegay, Kiyoshi Sugimoto, Caroline Di Benedetto, Christoph A Fux, Philip E Tarr, Roger Kouyos, Hansjakob Furrer, Gilles Wandeler, Swiss HIV Cohort Study

**Affiliations:** 1 Department of Infectious Diseases, Bern University Hospital, University of Bern, Bern, Switzerland; 2 Division of Infectious Diseases and Hospital Epidemiology, University Hospital Zurich, University of Zurich, Zurich, Switzerland; 3 Division of Infectious Diseases and Hospital Epidemiology, University Hospital Lausanne, Lausanne, Switzerland; 4 Division of Infectious Diseases and Hospital Epidemiology, University Hospital Geneva, Geneva, Switzerland; 5 Division of Infectious Diseases and Hospital Epidemiology, University Hospital Basel, University of Basel, Basel, Switzerland; 6 Cantonal Hospital, St. Gallen, Switzerland; 7 Regional Hospital, Lugano, Switzerland; 8 Cantonal Hospital Aargau, Aargau, Switzerland; 9 Cantonal Hospital Baselland, Bruderholz, University of Basel, Basel, Switzerland; 10 Institute of Social and Preventive Medicine, University of Bern, Switzerland

**Keywords:** HIV infection, late presentation, self-reported reasons, Switzerland, HIV testing, success to care

## Abstract

**Introduction:**

Late presentation to HIV care leads to increased morbidity and mortality. We explored risk factors and reasons for late HIV testing and presentation to care in the nationally representative Swiss HIV Cohort Study (SHCS).

**Methods:**

Adult patients enrolled in the SHCS between July 2009 and June 2012 were included. An initial CD4 count <350 cells/µl or an AIDS-defining illness defined late presentation. Demographic and behavioural characteristics of late presenters (LPs) were compared with those of non-late presenters (NLPs). Information on self-reported, individual barriers to HIV testing and care were obtained during face-to-face interviews.

**Results:**

Of 1366 patients included, 680 (49.8%) were LPs. Seventy-two percent of eligible patients took part in the survey. LPs were more likely to be female (*p*<0.001) or from sub-Saharan Africa (*p*<0.001) and less likely to be highly educated (*p*=0.002) or men who have sex with men (*p*<0.001). LPs were more likely to have their first HIV test following a doctor's suggestion (*p*=0.01), and NLPs in the context of a regular check-up (*p*=0.02) or after a specific risk situation (*p*<0.001). The main reasons for late HIV testing were “did not feel at risk” (72%), “did not feel ill” (65%) and “did not know the symptoms of HIV” (51%). Seventy-one percent of the participants were symptomatic during the year preceding HIV diagnosis and the majority consulted a physician for these symptoms.

**Conclusions:**

In Switzerland, late presentation to care is driven by late HIV testing due to low risk perception and lack of awareness about HIV. Tailored HIV testing strategies and enhanced provider-initiated testing are urgently needed.

## Introduction

In Europe, over 50% of HIV-positive patients are late presenters (LPs), defined as individuals presenting for HIV care with a CD4 cell count below 350 cells/µl and/or an AIDS-defining event (ADE) [1, 2]. Late presentation to care is associated with increased morbidity, mortality and poor treatment outcomes [3–5]. From a public health perspective, it contributes to new HIV infections through individuals who are unaware of their status [6] and causes avoidable healthcare costs [7]. Late HIV diagnosis through late HIV testing has been shown to be the main driver of late presentation to care; delayed presentation after a positive test is considered less important [2, 8].

Numerous studies, including from the Swiss HIV Cohort Study (SHCS) [9], have described demographic and structural risk factors associated with late presentation to HIV care, namely increased age, heterosexuality, low socio-economic status, low literacy, high-prevalence country of origin and the presence of logistic barriers [4]. Missed opportunities for HIV testing, especially in patients presenting with suggestive symptoms or indicator diseases, may also be an important driver [10, 11]. However, relatively little is known about individual self-reported reasons for late presentation. A recent systematic review identified only three studies that statistically evaluated psychosocial determinants of late presentation, two of them from Latin America [12]. In these small studies, low risk perception, stigma, fear and psychosocial distress were identified as potential sources of delayed testing or limited health-seeking behaviour [13–15]. These findings may differ by geographic region and health system and need to be confirmed in larger studies.

Despite increasing awareness of the main demographic and clinical drivers of late presentation and existing initiatives to address high-risk populations, late presentation remains a significant problem even in settings with good access to healthcare. In order to design and implement efficient strategies to tackle this problem, our understanding of the individual reasons for late presentation must be improved. We aimed to assess the prevalence of late presentation to HIV care in Switzerland, a country with mandatory health insurance for all official residents, and to identify related risk factors, including structural, behavioural and psychological barriers to HIV testing and linkage to care.

## Methods

### Swiss HIV Cohort Study

The SHCS (www.shcs.ch) is a prospective cohort study with ongoing enrolment of HIV-positive adults in Switzerland. It has remained representative of the HIV patient population since its inception in 1988 and currently covers at least 56% of the cumulative number of HIV-positive individuals the Swiss public health authorities have been notified about, 71% of patients living with AIDS and 75% of those receiving antiretroviral therapy (ART) [16]. Detailed information on demographics, mode of HIV acquisition, risk behaviour, clinical events, co-infections and treatment is collected at registration and then at six-month intervals. Local ethical committees of all participating study sites have approved the study and written informed consent is obtained from all participants.

### Study population and definitions

All adult patients newly included in the SHCS between 1 July 2009 and 30 June 2012 were analyzed. In line with the consensus statement of the European Late Presenter Consensus working group [1] we defined late presentation to HIV care as having a first CD4 T-cell count <350 cells/µl and/or an ADE within three months of presentation. The patients who did not belong to this group were considered as non-late presenters (NLPs). To describe specific patterns of late presentation to care, we additionally sub-classified late presentation into two groups according to the definitions proposed by Kozak *et al*.: “late HIV diagnosis” (or “late HIV testing”) was defined as having a CD4 cell count below 350 cells/µl within three months of HIV diagnosis and “delayed presentation for HIV care” as having a delay of more than three months between the first positive HIV test and subsequent outpatient medical visit [8]. Patients with known acute HIV infection at time of presentation were classified as NLPs regardless of initial CD4 cell count. All participants were included in the analyses of demographic and clinical predictors of late presentation for care. Additionally, all LPs as well as all NLPs from tertiary-care hospitals (control group) were asked to participate in a questionnaire-based survey on the individual HIV testing circumstances and reasons for late presentation to care.

### Questionnaire

To identify individual barriers to HIV diagnosis and care, we designed a paper-based questionnaire to be completed by all study patients agreeing to participate. Data were obtained through face-to-face interviews using a standardized questionnaire in German, French or English. Interviews were conducted by the treating physician or attending study nurse at all but three study sites (Geneva, Lausanne and Aarau, where questionnaires were completed by the patients themselves and then discussed with a physician or study nurse). For patients not fluent in any of the above languages, the questionnaire was completed with the aid of a translator. The questionnaire was based partially on a document from the Denmark initiative (courtesy of Prof. Jens Lundgren, Copenhagen HIV Programme, www.hiv-danmark.dk/fileadmin/user_upload/hiv-danmark/pdf/Late_presenters-FINAL.pdf) and was adapted to Swiss specificities. It included 43 questions on reasons for and circumstances of HIV testing, behavioural risk factors, patient awareness and knowledge of HIV, presence of symptoms and missed opportunities for HIV testing during the 12 months prior to referral for HIV care. The final part of the questionnaire was dedicated to LPs and assessed individual reasons for late testing and delayed presentation to care. When applicable, reasons for not completing the questionnaire were documented. An initial evaluation of the questionnaire data was performed in the context of a pilot study in Zurich [17]. This report did not include data from interviews with patients but assessed the relevance of the questions in a chart review.

### Statistical analyses

Differences in demographic, behavioural and clinical characteristics between LPs and NLPs were assessed using the Mann-Whitney and chi-square tests for continuous and categorical variables, respectively. Education level was defined as high if tertiary education was completed. Survey data from paper-based questionnaires were managed with REDCap (Research Electronic Data Capture) electronic data capture tools (www.redcap.vanderbilt.edu/) [18]. The most frequent reasons for testing were described in percentages and compared between LPs and NLPs using the chi-square test. Finally, we evaluated the main self-reported reasons for late presentation to care among LPs. All statistical analyses were performed with Stata 12.1 (StataCorp 2012, Stata Statistical Software, College Station, TX, USA).

### Sensitivity analysis

Patients with an unrecognized acute HIV infection may have a low CD4 cell count or an ADE at presentation. In order to avoid misclassification of these patients into the LP category, we repeated our analyses after excluding patients with an acute infection according to the ambiguity score, recently described by Kouyos *et al*. [19] and subsequently validated [20, 21]. This score provides information on the individual duration of HIV infection by determining the fraction of ambiguous nucleotides in the viral pol sequences and helps classify primary HIV infections. Genetic information to perform these analyses was derived from the genotypic resistance tests routinely performed at SHCS enrolment.

## Results

### Study population and baseline characteristics of LPs

**Figure 1:**
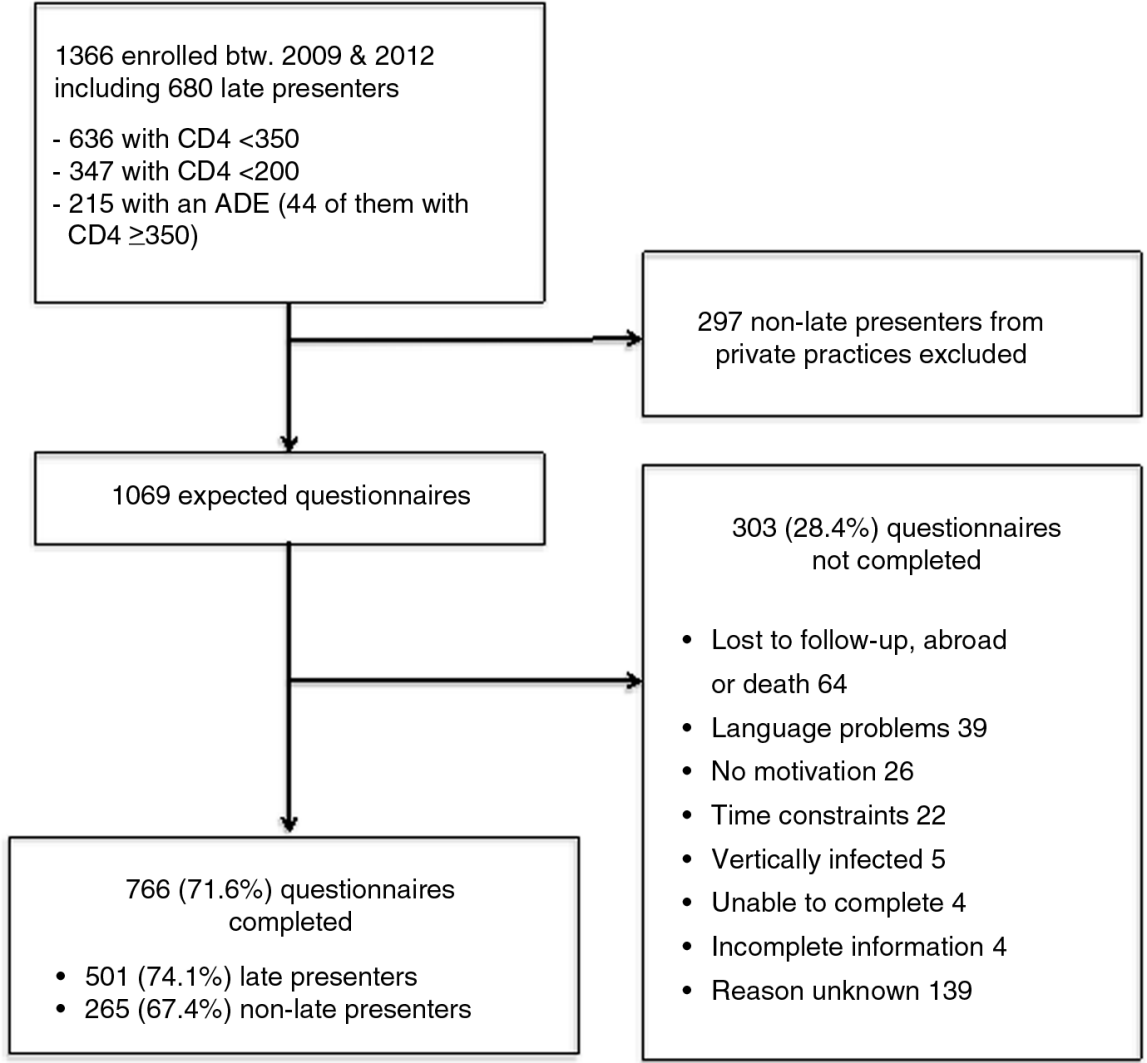
Flow chart of late presenter survey. ADE, AIDS-defining event.

During the study period, 1366 patients were enrolled in the SHCS, of whom 680 (49.8%) were LPs (Figure 1).

Among LPs, 636 (93.5%) had a CD4 count below 350 cells/µl and 215 (31.6%) an ADE; 347 (51.0%) had a CD4 count below 200 cells/µl. Forty-four of 215 patients with an ADE presented with a CD4 count of ≥350 cells/µl. The most common ADEs were *Pneumocystis jirovecii pneumonia* (65 cases, 30.2%), pulmonary tuberculosis (22 cases, 10.2%) and Kaposi's sarcoma (22 cases, 10.2%). Thirty-four individuals with a known primary infection, presenting with a CD4 count below 350 cells/µl or an ADE at diagnosis, were reclassified as NLPs. The proportion of LPs differed slightly between the SHCS centres, ranging from 44.4% in Zurich to 58.6% in Basel. Compared to NLPs, LPs were more likely to be male and heterosexual (29.1% vs. 18.6%) or female (30.0% vs. 20.0%) and to originate from sub-Saharan Africa (18.6% vs. 10.1%) or Asia (7.5% vs. 2.5%). NLPs were more likely to be men who have sex with men (MSM) (61.4% vs. 40.9%) and to have high-level education (43.4% vs. 35.6%) (Table 1).

**Table 1:** Demographic characteristics of late and non-late presenters

	Late presenters	Non-late presenters	
			
	*N*=680 (49.8%)	*N*=686 (50.2%)	*p*
Demographic group (%)			<0.001
MSM	278 (40.9)	421 (61.4)	
Non-MSM male	198 (29.1)	128 (18.6)	
Female	204 (30.0)	137 (20.0)	
Median age in years (IQR)	40.6 (32.7–48.4)	38.2 (31.0–45.4)	<0.001
Median first CD4 count in cells/µl (IQR)	195 (88–286)	511 (417–663)	<0.001
Region of origin (%)			<0.001
South + Northwest Europe	435 (64.0%)	515 (75.3%)	
Sub-Saharan Africa	126 (18.6%)	69 (10.1%)	
South + East Asia	51 (7.5%)	17 (2.5%)	
Other	67 (9.9%)	83 (12.1%)	
High-level education (%)	242 (35.6%)	297 (43.4%)	0.002

IQR, interquartile range; MSM, men who have sex with men.

### Questionnaire completion

Of 1366 patients enrolled, 297 NLPs from private practices were excluded from the survey for logistical reasons (Figure 1). Of the 1069 remaining patients, 766 (71.6%) completed the questionnaire. The main reasons for not completing the questionnaire are shown in Figure 1. Although patients who completed the questionnaire were slightly older (median age 40.5 vs. 38.4, *p*=0.02) and comprised more individuals of European origin (71.0% vs. 59.0%, *p*<0.01) than those who did not complete the questionnaire (Supplementary Table 1), there was no difference in the proportion of late presentation to care between these two groups (64.9% vs. 59.1%, *p*=0.08).

### Behavioural and clinical differences between late and NLPs

Compared to NLPs, LPs were more likely to be diagnosed with HIV during hospitalization (21.8% vs. 8.8%, *p*<0.01) and less likely to be aware of the place of infection (23.1% vs. 15.5%, *p*<0.01) or to remember a specific risk situation that could have led to infection (33.9% vs. 50.6%, *p*<0.01) (Table 2).

**Table 2:** Behavioural and clinical determinants of late presentation to care according to questionnaire data

	Late presenters	Non-late presenters	Total	
		
	(*N*=501)	(*N*=265)	(*N*=766)	*p*
First positive test during hospitalization (%)	104 (21.8)	22 (8.8)	126 (17.3)	<0.001
Place of infection (%)				<0.001
Switzerland	193 (40.6)	138 (55.4)	331 (45.7)	
Abroad	164 (34.5)	76 (30.5)	240 (33.1)	
Unknown	119 (25.0)	35 (14.1)	154 (21.2)	
Remembers specific risk situation (%)	160 (33.9)	126 (50.6)	286 (39.7)	<0.001
Stable relationship (%)^a^	288 (60.4)	133 (52.8)	415 (57.8)	0.05
Occasional sex partners (%)^a^	242 (51.3)	154 (61.9)	396 (54.9)	0.01
Inconsistent condom use (%)^a^	331 (79.4)	173 (75.6)	504 (78.0)	0.26
Symptoms^b^				
At least one symptom	366 (73.3)	177 (66.8)	544 (71.0)	0.06
Fatigue	175 (34.9)	71 (26.8)	246 (32.1)	0.02
Fever	123 (24.6)	73 (27.6)	196 (25.6)	0.37
Weight loss	142 (28.3)	26 (9.8)	168 (21.9)	<0.001
Respiratory infection	102 (20.4)	46 (17.4)	148 (19.3)	0.32
Skin lesions	91 (18.2)	28 (10.6)	119 (15.5)	0.01
Diarrhoea	85 (17.0)	34 (12.8)	119 (15.5)	0.13
Lymphadenopathy	73 (14.6)	45 (17.0)	118 (15.4)	0.38
Oral lesions	63 (12.6)	19 (7.2)	82 (10.7)	0.02
Muscle pain	45 (9.0)	24 (9.1)	69 (9.0)	0.97
At least one of the following symptoms: fatigue, weight loss, oral or skin lesions	254 (50.7)	102 (38.5)	356 (46.5)	0.001
Had a GP at time of diagnosis	315 (66.2)	173 (68.9)	488 (67.1)	0.45
Consultation for symptoms	234 (74.8)	108 (73.0)	342 (74.2)	0.68

GP: general practitioner.

a During the six months before diagnosis;

b during the 12 months before diagnosis;

Although the majority of patients had a stable relationship at time of diagnosis, 51.3% of LPs and 61.9% of NLPs had occasional sexual partners and over three-quarters of them reported inconsistent condom use. Of all patients, 71% had symptoms during the 12 months prior to diagnosis. This proportion was slightly higher in LPs compared to NLPs (73.3% vs. 66.8%, *p*=0.06). The most common symptoms were fatigue, fever and weight loss, each of which affected over 20% of the study population. Fatigue, weight loss and skin or oral lesions were more common among LPs (all *p*<0.05) (Table 2). Overall, 50.7% of LPs versus 38.4% of NLPs (*p*<0.01) exhibited at least one of these four manifestations. Of the symptomatic patients, 74.2% sought medical care because of symptoms, and over two-thirds had a general practitioner (GP) at the time of HIV diagnosis.

### Reasons for HIV testing

**Figure 2:**
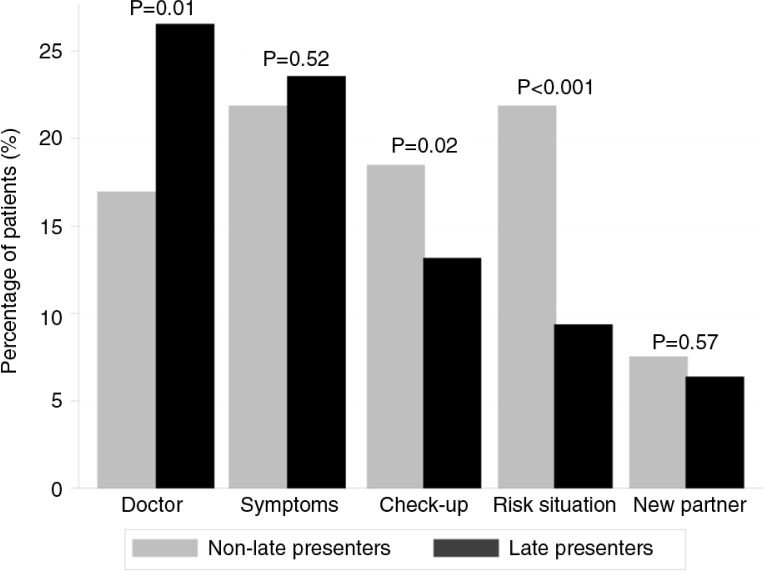
Main reasons for HIV testing among 501 late presenters and 265 non-late presenters. (Reason explanations: “doctor,” tested after doctor's suggestion; “symptoms,” tested because of relevant symptoms; “check-up,” tested in the context of a regular check-up; “risk situation,” tested after experiencing a risk situation; “new partner,” tested after starting a new relationship.)

The most frequent reasons for HIV testing are shown in Figure 2.

“Doctor's suggestion” was the most frequent overall (*n*=178, 23.2%), followed by “symptoms” (*n*=176, 23.0%), “regular check-up” (*n*=115, 15.0%) and “specific risk situation” (*n*=105, 13.7%). Few patients had their first positive test in the context of a pregnancy (*n*=30, 4.0%) or because of a positive partner (*n*=36, 4.7%). Similar proportions of LPs and NLPs reported having undergone their first positive HIV test due to relevant symptoms or because they had a new partner. However, LPs were significantly more likely to test following a doctor's suggestion (provider-initiated testing) compared to NLPs. In contrast, NLPs tested more often because of a specific risk situation or in the context of a regular check-up (Figure 2).

### Reasons for late HIV testing

**Figure 3:**
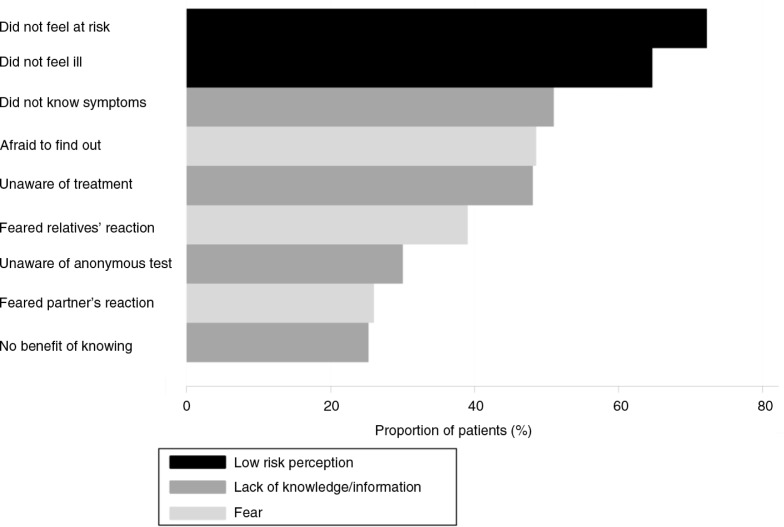
Reasons for late HIV testing among 236 late presenters.

Only 8.9% of patients reported waiting more than three months after their HIV diagnosis before presenting for medical care (delayed presentation). However, of the 501 LPs who completed the questionnaire, 236 (47.1%) stated that they realized they had not tested early enough. The most frequent self-reported reasons for testing late in these patients are summarized in Figure 3.

The majority of these individuals (72.3%) did not feel they were at risk of being infected with HIV and most of them (64.7%) did not test because they did not feel ill. Reasons linked to the lack of knowledge about HIV were also prevalent: many patients did not know the symptoms of HIV infection (51%) or were not aware of treatment (48%) or anonymous testing possibilities (30%). One-quarter thought there was no benefit in knowing their HIV status. Finally, reasons related to fear were also important: 48.6% were afraid to find out about a possible HIV infection, 39% feared their relatives’ reactions and 26.0% their partner's reactions.

### Sensitivity analysis

Among the LP group, 298/501 individuals (59.5%) had an ambiguity score available. Of these, 80 (27.5%) had a low score, suggesting recent infection. Excluding these patients from the analyses did not significantly alter the proportion of LPs and NLPs diagnosed during hospitalization, being aware of the place of infection or specific risk situations or being symptomatic before HIV diagnosis. The main reasons for HIV testing and the causes for late testing in LPs were also comparable to the main analyses (data not shown).

## Discussion

Despite the widespread availability of ART and good access to medical care, late presentation to care remains one of the biggest challenges in the management of HIV infection in high-income countries. Nearly half of the 1366 patients in our study were LPs. This trend was driven mainly by late HIV testing due to low risk perception and lack of awareness of HIV transmission, symptoms and treatment. LPs were more likely to be heterosexual, originating from sub-Saharan Africa and to have undergone the first positive test during a hospitalization or upon a doctor's suggestion. In contrast, NLPs, with a majority of MSM, more often had their first positive test during a regular check-up or in the context of a specific risk situation. These results illustrate the differences between LPs and NLPs in terms of demographic and behavioural characteristics, as well as HIV risk perception, and underline the need for targeted public health and communication strategies to enhance HIV testing in populations at high risk of late presentation.

The proportion of LPs observed in our study (49.8%) was similar to recent studies from Denmark [11] and Germany [22], but marginally lower than that reported in COHERE, a large European HIV cohort collaboration (53.8%) [2]. As there was a decrease in LP prevalence from 57.3% in 2000 to 51.7% in 2010/2011 in COHERE, our result could be explained by a slow, general improvement in early HIV testing and care in Europe. This hypothesis is also strengthened by the comparison of our results with previous data from the SHCS [9]: between 1998 and 2007, 31% of patients presented with CD4 <200 cells/µl and/or AIDS, whereas this proportion was 19% during our study period (2009 to 2012). The variability of the proportion of LPs between SHCS sites was driven potentially by differences in patient demographic characteristics: Zurich, for instance, with the lowest LP prevalence, had the highest proportion of MSM. In line with most published studies, the main demographic factors associated with late presentation to HIV care in our study were heterosexuality, female sex, increased age, low education and sub-Saharan African origin.

To understand the principal determinants of late presentation to care, it is necessary to look beyond patient demographic characteristics. Among the few studies that have described structural and social predictors of late presentation, Delpierre and colleagues showed that late presentation in France was associated with “living as part of a couple with children” [5]. The finding that LPs in the SHCS were also more likely to live in a stable partnership compared to NLPs confirms that late presentation is more prevalent in patient categories not traditionally considered high risk for HIV infection. HIV risk perception in our study was lower among LPs than NLPs: LPs were more often diagnosed during hospitalization and less likely to remember the place of infection or a specific situation that may have led to the infection. There were also large differences in the main reasons for HIV testing between LPs and NLPs: LPs were more likely to perform the first HIV test upon a doctor's suggestion whereas NLPs tested more regularly during routine check-ups or after specific risk situations. Thus, the description of the context in which patients underwent their first positive HIV test underlined the lack of awareness of HIV risk in LPs and the importance of provider-initiated HIV testing in this population.

Previous reports have shown that late presentation to HIV care was driven almost exclusively by late HIV testing and that delayed presentation after a positive HIV test was was true for only a fraction of these patients [2]. Our data confirm these findings: 9% of LPs reported having waited over three months to seek medical care after their first positive test. In contrast, approximately one-half of LPs admitted to having performed their first HIV test too late. Patterns of late presentation to care might differ in countries where access to HIV medical care is difficult for some populations. In a recent study from New York City of 1928 patients with newly diagnosed HIV infection, only 63.7% were linked to medical care within three months after their positive test [23]. The two most common self-reported reasons for late HIV testing among LPs in our study were “I did not feel at risk” and “I did not feel ill.” As reported in a previous study from South America [13], this shows that low perception of HIV risk was also a major driver of late presentation to care in our setting. The fact that many LPs recalled having relevant symptoms and a GP at the time of HIV diagnosis but still did not feel at risk of infection also highlights differences in health perception and health-seeking behaviour, which may vary with origin and socio-cultural background [24]. Finally, reasons relating to lack of knowledge regarding HIV infection in our study were reported more frequently than those linked to fear and discrimination, which might be more prevalent in resource-limited settings [25].

To our knowledge, this is the first study to assess demographic, socio-economic and behavioural risk factors for late presentation to HIV care in a nationwide cohort. Furthermore, our detailed questionnaire allowed identification of the circumstances in which patients performed their first positive HIV test and the main reasons for late HIV testing. The combination of routine HIV care data and information from a dedicated survey allowed us to create a unique dataset dedicated to the study of the main determinants of late presentation to care. However, we acknowledge several limitations. The group of patients who presented late according to the definition proposed by European Late Presenter Consensus working group [1] included a number of individuals with primary or early HIV infection. We addressed this challenge in two ways. First, we reclassified 34 patients with low CD4 counts and known primary infection into the NLP category. Second, we repeated our main analyses after excluding individuals with possible early infection according to their ambiguous nucleotide score [19], a measure that was shown to identify false LP in a previous analysis from a subset of the SHCS [17]. This step afforded a more accurate picture of late presentation to care in our study compared to previous reports. Another limitation was the survey participation rate, with approximately one-quarter of eligible patients declining or being unable to complete the questionnaire. Against this, the baseline characteristics of patients with and without questionnaire data were similar, suggesting that incomplete participation did not bias our observations. Finally, as with any survey, reporting and recall biases may have influenced the data collected.

In summary, in this nationwide HIV cohort, late presentation to care was more frequent in patients from sub-Saharan Africa and Asia and in those not traditionally considered at high risk. Insufficient awareness regarding HIV transmission and symptoms were the most important drivers of late testing. Given the economic and health consequences of late presentation to care, healthcare workers and public health authorities need to improve HIV testing strategies and elaborate methods for increasing testing during early infection in specific populations, notably patients from sub-Saharan Africa and married couples. To date, there is still no free and anonymous HIV testing platform in Switzerland, and specific health structures have been developed mostly for MSM. Most LPs have a GP, experience symptoms during the year preceding the HIV diagnosis and consult a doctor for these symptoms. These missed opportunities for earlier diagnosis need to be addressed by enhancing provider-initiated testing. Primary care and specialist physicians need to increase HIV testing among their patients if earlier diagnosis and linkage to care are to be achieved.

## Supplementary Material

Reasons for late presentation to HIV care in Switzerland
