# Supplementary material for: Reasons for late presentation to HIV care in Switzerland
Source: J Int AIDS Soc. 2015 Nov 18;18(1):20317. doi: 10.7448/IAS.18.1.20317 (PMC4653319; doi:10.7448/IAS.18.1.20317)
Supplement: Reasons for late presentation to HIV care in Switzerland [file JIAS-18-20317-s001.pdf]

Supplementary Table 1: Comparison of baseline characteristics between survey participants and non-participants

	**Questionnaire available** (N=766)	**Questionnaire unavailable** (N=303)	**Total** (N=1,069)	**P-value**
Female (%)	190 (24.8)	91 (30.0)	281 (26.3)	0.08
Median age in years (IQR)	40.5 (32.4 - 47.8)	38.4 (31.5 - 45.9)	40.0 (32.2-47.4)	0.02
Median first CD4-count in cells/µl (IQR)	300 (171-455)	280 (144-449)	287 (154-449)	0.25
MSM transmission group (%)	374 (48.8)	137 (45.2)	511 (47.8)	0.63
Region of origin (%)				<0.01
S and NW Europe	542 (71.0)	179 (59.0)	721 (67.6)	
Sub-Saharan Africa	117 (15.3)	52 (17.2)	169 (15.9)	
S and E Asia	38 (5.0)	24 (7.9)	62 (5.8)	
Other	66 (8.7)	48 (15.8)	410 (38.4)	
High-level education (%)	298 (39.0)	112 (37.0)	410 (38.4)	0.19

IQR: interquartile range, MSM: men who have sex with men; S: South; NW: Northwest; E: East

Supplementary Table 2: Behavioral and clinical determinants of late presentation to care according to questionnaire data, after exclusion of patients with low ambiguity score

	Late Presenters	Non-Late Presenters	Total	P-value
First positive test during hospitalization (%)	93 (22.1)	22 (8.3)	115 (16.8)	<0.001
Place of infection (%)				<0.001
Switzerland	153 (38.5)	138 (55.4)	291 (45.1)	
Abroad	138 (34.8)	76 (30.5)	214 (33.1)	
Unknown	106 (26.7)	35 (14.1)	141 (21.8)	
Remembers specific risk situation (%)	130 (33.1)	126 (50.6)	256 (39.9)	<0.001
Stable relationship (%) *	243 (61.2)	133 (52.8)	376 (57.9)	0.03
Occasional sex partners (%)*	194 (49.5)	154 (61.9)	348 (54.3)	0.002
Inconsistent condom use (%)*	278 (80.3)	173 (75.6)	451 (78.4)	0.17
Symptoms **				
At least 1 symptom	304 (72.2)	177 (66.8)	481 (70.1)	0.13
Fatigue	146 (34.7)	71 (26.8)	217 (32.6)	0.03
Fever	102 (24.2)	73 (27.6)	175 (25.5)	0.33
Weight loss	118 (28.0)	26 (9.8)	144 (21.0)	<0.001
Respiratory infection	88 (20.9)	46 (17.4)	134 (19.5)	0.25
Skin lesions	83 (19.7)	28 (10.6)	111 (16.2)	0.002
Diarrhea	67 (15.9)	34 (12.8)	101 (14.7)	0.27
Lymphadenopathy	61 (14.5)	45 (17.0)	106 (15.5)	0.38
Oral lesions	58 (13.8)	19 (7.2)	77 (11.2)	0.01
Muscle pain	42 (10.0)	24 (9.1)	66 (9.6)	0.69
At least 1 of fatigue, weight loss, oral- or skin lesions	211 (50.1)	102 (38.5)	313 (45.6)	0.003
Had a GP at time of diagnosis	257 (64.7)	173 (68.9)	430 (66.4)	0.27
Consultation for symptoms	190 (75.1)	108 (72.8)	298 (74.3)	0.64

* During the 6 months before diagnosis

** During the 12 months before diagnosis

GP: general practitioner
